# Optimization of the scale-up production process for high-yield laccase from white-rot fungi

**DOI:** 10.3389/fbioe.2025.1631687

**Published:** 2025-08-15

**Authors:** Yaping Ma, Minchang Liu, Ruifang Gu, Rongya Zhang, Xiaomei Ji, Juan Zhang, Wu Wen, Zheng Peng

**Affiliations:** ^1^ Technology Center, China Tobacco Sichuan Industrial Co., Ltd., Chengdu, China; ^2^ Key Laboratory of Industrial Biotechnology, Ministry of Education, School of Biotechnology, Jiangnan University, Wuxi, China; ^3^ Science Center for Future Foods, Jiangnan University, Wuxi, China

**Keywords:** white-rot fungus, Ganoderma lucidum, laccase, response surface methodology, fermentation optimization

## Abstract

**Introduction:**

Laccase exhibits significant applications in food additives, wastewater treatment, and biomass processing. Fungal laccase exhibits high activity, good stability, and excellent performance. However, scale-up production of high-yield laccase from fungi is challenging. This study aimed to identify crucial factors affecting enzyme production and analyze the enzymatic properties of laccase during fermentation.

**Methods:**

A laccase-producing white-rot fungus was used for fermentation process optimization in 200 L and 1200 L fermenters. The Plackett–Burman design revealed three significant influencing factors: temperature, aeration ratio, and agitation speed. The steepest ascent experiment was used to approximate the maximum response region, followed by the establishment of a regression model between experimental factors and laccase activity using the Box–Behnken response surface methodology and optimal fermentation condition selection.

**Results:**

The optimal conditions for laccase production by *Ganoderma lucidum* fermentation were 30°C temperature, 0.66 aeration ratio, and 100 rpm agitation speed, achieving a maximum laccase activity of 214,185.2 U/L. Dissolved oxygen (DO) was a crucial factor for high laccase yield, and its maintenance at a high level in the fermentation system significantly enhanced the enzyme activity. Fermentation batches with varying laccase production levels exhibited a trend of decreasing pH and a subsequent increase in the mid-to-late fermentation stages. With decreased pH, the DO level started declining; when DO stabilized, the pH started to rebound, coinciding with the peak laccase activity, indicating a signal of fermentation endpoint in industrial production.

**Discussion:**

This study provides valuable theoretical and data support for the industrial production of laccase by fungi through optimized fermentation processes.

## 1 Highlights

• Crucial factors affecting laccase activity determined using Plackett–Burman design
• Enzymatic conditions of the crucial factors were optimized through Box–Behnken RSM
• The reduction of the agitation speed significantly increased laccase activity
• Laccase is an acidic enzyme, as indicated by the low optimal pH
• Dissolved oxygen is a crucial factor for high laccase yield

## 2 Introduction

Laccase (EC 1.10.3.2), a copper-containing polyphenol oxidase, possesses profound catalytic abilities for various compounds, including phenols, aromatic amines, and non-phenolic entities. This unique functional characteristic has prompted its extensive applications in energy development, bio-detection, biomass degradation, the papermaking industry, the food industry, and pollutant treatment (Mate and Alcalde, 2017; Kudanga et al., 2017; Zhou et al., 2021; Dong et al., 2023). The commercial laccase enzymes available in the market are relatively expensive. For instance, the 500 U/g laccase products from Shanghai Yuanye Biotechnology Co., Ltd. And Aladdin Reagent Co., Ltd. Are priced at approximately 279.2 $/g, while the 120 U/g laccase products cost around 13.96 $/g. The report titled “Global Laccase Market Size, Manufacturers, Supply Chain, Sales Channel and Clients, 2025–2031″reported by The statistics from QYResearch (Hengzhou bozhi) indicates that in 2024, the global market size of laccase was estimated at approximately US3.3million.ItisprojectedthatthemarketwillexpandtoaroundUS4.4 million by 2031, exhibiting a compound annual growth rate of 4.3% during the forecast period spanning from 2025 to 2031. Laccase is distributed in plants, insects, bacteria, and fungi, with microorganisms, particularly fungi, being the primary producers. Among them, white-rot fungi have been extensively studied (Han et al., 2023; Zhai et al., 2024). White-rot fungi rapidly grow on lignin substrates and secrete laccase, making low-cost lignocellulosic materials excellent substrates for fungal laccase production (Han et al., 2023). Lignin substrates such as corn cobs and cottonseed hulls, which are byproducts of agricultural production, are primarily used for laccase production by white-rot fungi, realizing the high-value utilization of agricultural waste (Carolina et al., 2022). In this study, the lignocellulosic material used was tobacco stem, a byproduct of tobacco processing that accounts for approximately 20%–30% of tobacco leaves, with an annual production of over 500,000 tons in China, representing a significant quantity with low economic value (Zhou et al., 2024). The use of tobacco stems for laccase production achieves effective waste utilization, and the produced laccase facilitates the enzymatic treatment of tobacco leaves to enhance their aroma and quality (Yu et al., 2017).

Common fermentation methods for laccase production by white-rot fungi include solid-state fermentation (SSF) and submerged fermentation (SmF) (An et al., 2021; Dávila-Giraldo et al., 2022; Sodhi et al., 2024). SSF simulates the natural growth conditions of white-rot fungi, facilitating enzyme production; however, it complicates downstream extraction (Chmelova et al., 2022). The fermentation conditions of SmF are easier to adjust than those of SSF, and laccase is secreted into the liquid medium, which benefits batch stability in industrial production and downstream extraction (An et al., 2020). Numerous studies exist on the preparation of high-yield laccase through SmF in small laboratory fermenters. For example, Schneider et al. (2018) optimized the carbon and nitrogen sources for the white-rot basidiomycete *Marasmiellus palmivorus* and scaled up to a 5-L reactor, achieving a laccase activity of 3420 U/mL after 186 h of cultivation. Similarly, Li et al. (2015) optimized the medium for the fungus Trametes sp. LS-10C and scaled up to a 10-L fermenter, obtaining a laccase activity of 873.82 U/mL on the ninth day. In addition, Songulashvili et al. (Songulashvili et al., 2015) fermented the white-rot fungus Cerrena unicolor C-139 in a 120 L fermenter, achieving a laccase activity of 416.4 U/mL on the 12th day of fermentation. Moreover, Songulashvili et al. (2016) achieved high laccase production of 200,900 U/L within 7 days through a 50-L scale-up of SmF.

While laccase activities may reach high levels in laboratory shake flasks and small fermenters, the fermentation cycle is long, and achieving short-term laccase production remains challenging. Wang et al. (2016) used vanillic acid as an inducer and achieved a laccase activity of 785.12 U/L after 4 days of fermentation in a 200 L bioreactor. This scale was larger than those in the aforementioned studies, and the fermentation time was shorter; however, the enzyme activity was not high. Pilot-scale or industrial-scale fermentation for laccase production usually results in low enzyme activities and has been less studied. Concerning scale-up production, Liu et al. (2016) achieved a maximum laccase yield of 80 U/mL using a 5-ton scale high-density cultivation method; however, this was significantly lower than the 240 U/mL yield in a 5-L small-scale fermenter under the same conditions. However, this study was pioneering in terms of such a scale-up fermenter. Although researchers have optimized the SmF of white-rot fungi, industrial laccase production faces many challenges, such as low efficiency, low stability, and high prices (Khatami et al., 2022).

Scaling up from the laboratory to industrial production may render laboratory process parameters inapplicable. Long fermentation times cause contamination, impeded microbial growth and reproduction, and low efficiency, particularly as filamentous fungi commonly produce foam in scale-up fermenters. Therefore, optimizing the fermentation conditions of white-rot fungi based on pilot- and industrial-scale operations, shortening the fermentation time, and achieving high-efficiency and high-yield laccase production are crucial.

This study aims to identify crucial factors affecting enzyme production and analyze the enzymatic properties of laccase during fermentation.

In this study, white-rot fungi were used as the carrier, and based on a 200 L fermenter, Plackett–Burman experiments were conducted to identify significant factors affecting enzyme production during fermentation. The steepest ascent experiment was used to approach the optimal range of crucial factors, and a Box–Behnken response surface methodology (RSM) experiment was used to establish an enzyme production model and obtain optimal fermentation parameters. Finally, the loading ratio was optimized in a 1200 L industrial fermenter, and the enzymatic properties of laccase were analyzed.

## 3 Materials and methods

### 3.1 Strains

The *Ganoderma lucidum* strain is preserved in our laboratory.

### 3.2 Medium

Potato Dextrose Broth (PDB): Biological reagent, Haibo Bio-tek; Glucose, MgSO_4_·7H_2_O, KH_2_PO_4_, Vitamin B1, NaOH, Citric Acid, K_2_HPO_4_, analytically pure, Sinopharm Chemical Reagent Co., Ltd.; Yeast Extract, Soy Peptone, Beef Extract Peptone, Corn Steep Liquor: Biological reagents, Sinopharm Chemical Reagent Co., Ltd.; Wheat Bran: Food grade, Qingdao Flour Processing Plant, Shandong Province; Antifoam agent: Deqing Kangle Fine Chemical Factory.

The laccase production medium formula for *G. lucidum*, optimized through single-factor experiments, Plackett–Burman design, and Box–Behnken response surface methodology in our laboratory, was yeast extract (3.0 g/L), corn steep liquor (20.0 g/L), wheat bran (31.6 g/L), tobacco stem powder (10.0 g/L), MgSO_4_·7H_2_O (1.0 g/L), KH_2_PO_4_ (1.1 g/L), vitamin B1 (0.1 g/L), and the medium was sterilized at 121°C for 30 min (Ma et al., 2023). This formula is used at shake flask and scale-up production stages.

### 3.3 Instruments and equipment

Fermenter (200 L): Huisen; 1200 L Fermenter: Fengze Bio-tech; Autoclave: FD100A model, Zhiwei Instrument Co., Ltd.; Clean Bench: BCM-1000A model, Suzhou Antai Air Technology Co., Ltd.; Horizontal Full-temperature Shaking Incubator: ZQWY-200V, Shanghai Zhichu Instrument Co., Ltd.; UV Spectrophotometer: 1810 model, Shanghai Puxi; Electronic Balance: JE3002GE model, Mettler.

### 3.4 Strain cultivation method

Preparation of inoculum: Inoculation of three to four mycelial plugs from a plate into 50 mL of a PDB medium in a 250 mL Erlenmeyer flask and incubation at 30°C and 150 rpm for 5 days. The mycelium was dispersed using glass beads for subsequent use.

Shake-Flask Fermentation for Enzyme Production: A fermentation medium (50 mL) was prepared in a 250 mL Erlenmeyer flask, followed by the addition of 3 mL of the prepared inoculum and incubation for 5 days at 30°C and 180 × g. The laccase activity was measured in the fermentation broth.

### 3.5 Laccase activity assay method

Enzyme activity was determined as follows: A 3 mL reaction mixture containing 0.5 mL of 2 mmol/L ABTS solution, 2 mL of 0.05 mol/L (pH 3) sodium phosphate-citric acid buffer, and 0.5 mL of crude enzyme solution (fermentation broth filtered through a 0.22 µm membrane) was incubated for 5 min at 45°C, and the optical density at 420 nm was measured (Babinskas and Matijosyte, 2025).

$$\text{Laccase activity}=\frac{10^{6}\times V_{total}\times A_{420}}{\varepsilon \times V_{enzyme}\times ∆t}$$

where:

ε: Coefficient, with a value of 3.6 × 10^4^ L/(mol × cm);

V_total_: Total volume of the laccase activity assay reaction mixture, in mL;

V_enzyme:_ Volume of enzyme solution added, in mL;

∆t: Reaction time, in min.

Each experiment was performed in triplicate, and laccase activity was measured thrice for each sample, with the average value taken.

### 3.6 Plackett–Burman experiment

The primary factors affecting laccase production by *G*. *lucidum* in a fermenter are fermentation temperature, aeration rate, agitation speed, and medium volume. Set a gradient range of 1.3–2 times and conduct experiments according to the Plackett Burman design to measure laccase activity at 96 h of fermentation.

### 3.7 Steepest ascent experiment design

Based on the key factors screened using the Plackett–Burman experiment, a certain step size was set, and the optimal range of the crucial factors was determined by measuring laccase activity.

### 3.8 Box–Behnken response surface methodology optimization

Response surface analysis was used to optimize the enzymatic conditions. Based on the crucial factors and optimal ranges determined using Plackett–Burman and steepest ascent experiments, a three-factor, three-level experimental design was adopted using the central composite design principle of Box–Behnken (Silva et al., 2022; Mengistu et al., 2024). The response variable Y represented laccase activity, and the response factors A, B, and C were the fermentation temperature, aeration rate, and agitation speed, respectively. Twelve experimental runs were designed. The Design-Expert 12 software was used to perform regression fitting on the experimental results in a table to establish a quadratic response surface regression model. The Design-Expert 12 software was used to draw response surface and contour plots of the interactions between the three factors. The relationships between pH, dissolved oxygen (DO), laccase activity, and the optimal endpoint of fermentation were analyzed for high, medium, and low enzyme-producing batches.

### 3.9 Optimization of fermentation conditions

Based on a 1200 L fermenter, the initial conditions for the experiment were set as follows: fermentation temperature 30°C, aeration rate 0.66, agitation speed 100 rpm, and antifoam agent addition 0.05%. Single-factor optimization was performed for antifoam agent type, aeration rate, agitation speed, and antifoam agent addition. Foam generation in the fermenter was observed through a sight glass, and enzyme activity was measured as an evaluation indicator.

### 3.10 Analysis of laccase enzyme activity characteristics

The enzyme activity changes of the crude enzyme solution at 50°C, 30°C, 18°C, and 4°C over different periods were measured to investigate the stability of laccase activity at different temperatures. The optimal reaction temperature of laccase was determined by setting different reaction temperatures (30°C, 40°C, 50°C, 60°C, and 70°C) between laccase and ABTS and measuring the corresponding enzyme activity. The optimal pH of laccase was determined by placing laccase in buffer solutions with pH values of 2.0, 3.0, 4.0, 5.0, 6.0, and 7.0 and measuring the enzyme activity. The residual enzyme activity was calculated with the highest enzyme activity in each group as 100%.

### 3.11 Statistical data analysis

Design-Expert 12 was used for experimental design and result analysis.

## 4 Results and discussion

### 4.1 Plackett–Burman experiment for enzyme production

The crucial factors affecting laccase production by *G*. *lucidum* in a fermenter were screened through the Plackett–Burman experiment. The experimental factors and levels are provided in Supplementary Table S1; and the experimental results and analysis are summarized in Table 1; Supplementary Table S2. The model was significant (*P* < 0.01) with a coefficient of determination of 0.999, indicating that the model has high fit and reliability. Among the factors, fermentation temperature (*P* < 0.01), aeration rate (*P* < 0.05), agitation speed (*P* < 0.01), and medium volume (*P* < 0.01) significantly affected laccase activity in the fermenter. All experimental batches with a medium volume of 0.6 had low enzyme activities not exceeding 2 × 10^4^ U/L and produced severe foam. Microscopic examination and plating results showed no contamination in the fermentation broth. The foam reduces oxygen exchange between the gas and liquid phases, causing decreased oxygen availability for *G. lucidum* in the fermentation broth, affecting its growth, reproduction, and metabolism (Beltrán-Flores et al., 2023). Therefore, subsequent response surface optimization was performed on fermentation temperature, aeration rate, and agitation speed based on a medium volume of 0.4. After optimizing these three parameters, the medium volume challenge, which limits production efficiency, will be addressed.

### 4.2 Enzyme production in the steepest ascent experiment

The fermentation temperature, ventilation ratio, and rotation speed were selected and analyzed sequentially with a certain step size in a downward direction (Table 2). The enzyme activity levels of experimental groups 2 and 3 were relatively high. Therefore, experimental groups 2 and 3 were chosen as the experimental range for the response surface methodology.

**TABLE 1:** Variance analysis results of the Plackett-Burman experiment.

Source of variance	Sum of squares	Estimation coefficient	P Value
model	4.756E+09		0.00**
temperature	1.662E+09	−13695	0.00**
Ventilation volume	7.045E+07	−2,884	0.03*
Rotational speed	6.064E+08	−7,747	0.00**
Inoculation amount	3.210E+07	1903	0.09
Medium volume	3.763E+10	−61021	0.00**
error	1.550E+07		
*R* ^2^	1.000		
R^2^_Adj_	0.999		

Note:*P*-values ≤0.05 were considered significant for all analyses performed and are indicated with an asterisk: *, *P* ≤ 0.05; **, *P* ≤ 0.01; ns, *P* > 0.05.

**TABLE 2:** Steepest ascent in terms of experimental results.

No.	Temperature (°C)	Ventilation volume (VVM)	Rotational speed (rpm)	Laccase activity (U/L)	Laccase activity SD (U/L)
1	34	1.0	120	109,538	3,005
2	32	0.8	100	150,317	4,281
3	30	0.6	80	165,453	4,093
4	28	0.4	60	95,166	3,376
5	26	0.2	40	46,307	2,619

### 4.3 Enzyme production in the Box–Behnken response surface experiment

Using Design-Expert 12 software for response surface analysis, a quadratic polynomial regression equation was obtained for the influence of various factors on laccase activity (Supplementary Tables S3, S4): $\text{Laccase activity}=-522.75A+14687.62B+39299.38C+4329.71\text{AB}-4329.71\text{AC}+4329.71\text{BC}+6123.14A^{2}$ .

The regression analysis of variance for the laccase production model from *G. lucidum* fermentation is provided in Table 3. The regression model was highly significant (*P* < 0.01), and the lack of fit was not significant (*P* > 0.05), indicating that the model was significant and had a good fit. This suggests that the model design is reasonable and highly reliable, with a regression coefficient *R* ^2^ = 0.990. The adjusted *R* ^2^ (R^2^Adj) was 0.962, indicating that the model explained 96.2% of the influence of factors on enzyme production changes. Therefore, the model was used to analyze and predict laccase production by *G. lucidum*. Analyzing the order of influence of various factors on enzyme production, ventilation ratio, rotation speed, fermentation temperature * ventilation ratio, fermentation temperature^2^, and ventilation ratio^2^ is significant. Based on the significance of *P*-values, the order of influence of factors was rotation speed > fermentation temperature^2^ > ventilation ratio > ventilation ratio^2^ > fermentation temperature * ventilation ratio.

**TABLE 3:** ANOVA of box-behnken.

Source of variance	Sum of squares	Estimation coefficient	P Value
model	2.162E+10	1.779 E+05	0.007**
A	2.186E+06	−522.75	0.875
B	1.726E+09	14,687.62	0.02*
C	1.236E+10	39,299.38	0.00**
A*B	8.250E+08	4,329.71	0.045*
A*C	3.622E+07	4,329.71	0.537
B*C	2.659E+08	4,329.71	0.156
A^2^	6.334E+09	6,123.14	0.003**
B^2^	1.053E+09	6,123.14	0.033*
C^2^	0		
Residual	2.250E+08		
Cor Total	2.184E+10		
*R* ^2^	0.990		
R^2^_Adj_	0.962		
S/N	19.5943		

Note:*P*-values ≤0.05 were considered significant for all analyses performed and are indicated with an asterisk: *, *P* ≤ 0.05; **, *P* ≤ 0.01; ns, *P* > 0.05.

**FIGURE 1:**
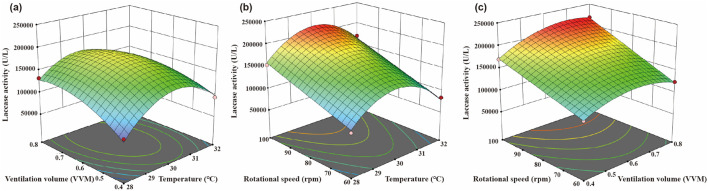
Interactive response map of various factors **(a)** Ratio of ventilation volume to temperature. **(b)** Ratio of rotational speed to temperature. **(c)** Ratio of rotational speed to ventilation volume.

Using Design Expert 12 software, response surface plots and contour maps were generated to illustrate the interactions among the three factors. When the ventilation ratio and agitation speed were held constant, the response value exhibited an initial increase followed by a decrease with increasing fermentation temperature (Figure 1). Similarly, when two factors were fixed, the response value trended upwards with increasing levels of ventilation ratio or agitation speed.

Based on the regression equation model, the predicted optimal conditions were a fermentation temperature of 30.2°C, a ventilation ratio of 0.66, and an agitation speed of 99.8 rpm, with the corresponding quadratic RSM predicting a laccase activity of 221,129.7 U/L. These conditions were adjusted to a fermentation temperature of 30 °C, a ventilation ratio of 0.66, and an agitation speed of 100 rpm according to practical production considerations. Under these optimal conditions, the average laccase activity from three parallel fermenter trials was 214,185.2 U/L, falling within a 5% deviation range, indicating a good fit between the model’s predicted and actual measured values.

**FIGURE 2:**
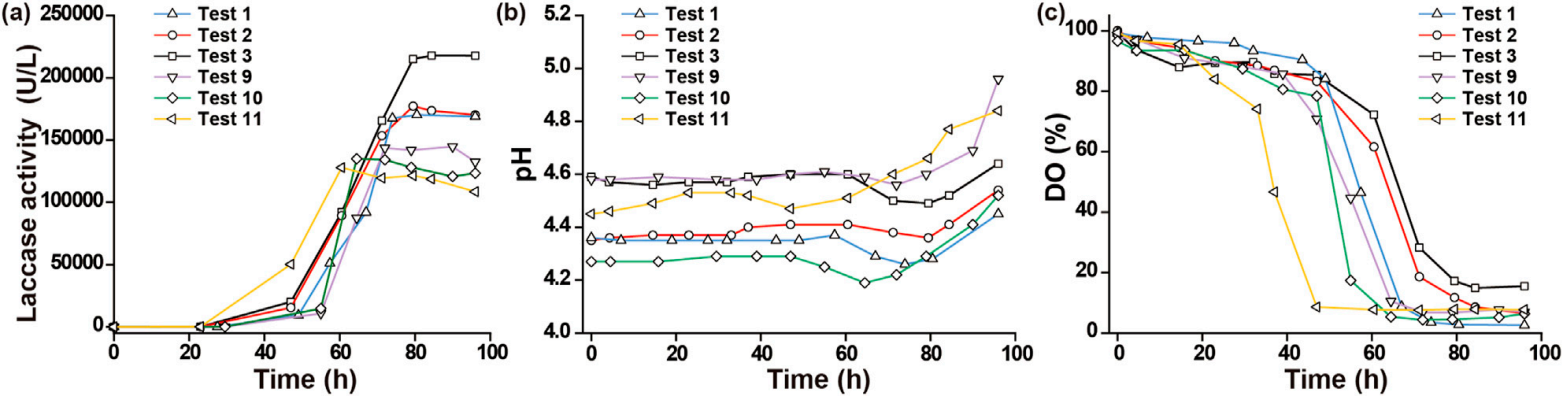
Laccase activity, pH, and dissolved oxygen (DO) of *Ganoderma lucidum* fermentation liquor at different enzyme production levels **(a)** Enzyme production curves of different fermentation batches. **(b)** pH variation curve of different fermentation batches. **(c)** DO variation curve of different fermentation batches.

Box–Behnken response surface test numbers 1, 2, 3, 9, 10, and 11 were selected, with test numbers 1, 2, and 3 representing the high laccase activity group, achieving laccase activities of 168,978 U/L, 170,197 U/L, and 217,724 U/L after 96 h of fermentation, respectively. Test numbers 9, 10, and 11 represented the low laccase activity group, with activities of 132,568 U/L, 123,463 U/L, and 108,550 U/L after 96 h, respectively. Figure 2 provides the changes in pH, DO, and enzyme activity over fermentation time for these six batches. The initial pH of the fermentation broth varied among batches owing to differences in condensate volume during sterilization and pH calibration errors. However, the pH of the fermentation broth at different laccase activity levels exhibited a trend of initial stability, followed by a decrease and a subsequent increase, consistent with the findings of Liu et al (Liu et al., 2013). Higher laccase activity was associated with a longer fermentation time corresponding to the lowest pH point. This may be attributed to the stable pH phase representing the growth and reproduction stages of *G. lucidum*, with a longer duration indicating better growth. The subsequent pH decrease marked the rapid laccase production stage, potentially owing to carbon and nitrogen source consumption by the fungus, causing acid production and pH decline. After the pH started increasing, laccase activity remained stable or even declined, suggesting that laccase production ceased at this point. The corresponding decrease in enzyme activity was caused by the instability of the laccase with respect to storage temperature and time. Thus, the highest laccase activity occurred when pH increased from its lowest level, serving as a signal for fermentation termination. Moreover, in the industrial production of laccase by *G. lucidum*, the timing of fermentation termination is determined by observing pH trends.

The DO of the fermentation broth at different laccase activity levels exhibited a trend of an initial slow decrease, followed by a rapid decrease before stabilization. The three groups with higher laccase activity levels corresponded to higher DO levels, indicating that DO facilitates laccase production by *G. lucidum*. A balance between DO and foam is crucial, as increasing the ventilation ratio and agitation speed enhances DO but may cause increased foam formation. Balancing these factors is essential to appropriately increase DO without generating foam. The graph curves revealed that the DO curves for batches with lower laccase activity levels shifted to the left, indicating an earlier onset of rapid DO decrease, which correlated with lower laccase activity. Comparing the pH, DO, and laccase activity curves, it is evident that the time points corresponding to the lowest pH began to increase, DO stabilized after an initial decrease, and laccase activity peaking was consistent.

### 4.4 Optimization of liquid-loading ratio in experimental settings

In industrial production processes, the liquid-loading ratio is a crucial indicator, with a higher ratio implying greater production efficiency. Mazumber et al. (Mazumder et al., 2008) have demonstrated that the volume of liquid loaded in a fermentation tank is a significant factor influencing laccase production, with a 20% reduction in liquid volume resulting in doubled laccase output. During microbial fermentation, the effects of protein-based surfactants, aeration conditions, and agitation cause foam formation in the fermentation broth. Foam leads to a decrease in the liquid-loading ratio, reduced oxygen transfer efficiency, increased risk of contamination, hindered bacterial respiration, and disrupted metabolism. Foam formation is a common challenge in the fermentation of filamentous fungi. In previous experiments, severe foam formation was observed when the liquid-loading ratio was 0.6. The observation of the foam formation pattern in each batch indicated that foam developed between 24 h and 48 h of fermentation and diminished by 48–54 h. This period is crucial for the growth, reproduction, and laccase production metabolism of *G. lucidum*. Using a 1200 L fermentation tank, optimizations were conducted focusing on the critical foam-forming period in terms of antifoam type, aeration rate, agitation speed, and antifoam dosage. Corn oil, polyether antifoam, and silicone oil antifoam were tested, resulting in foam reaching the top of the tank in all cases without contamination but with sparse microbial biomass and enzyme activities not exceeding 30,000 U/L.

**FIGURE 3:**
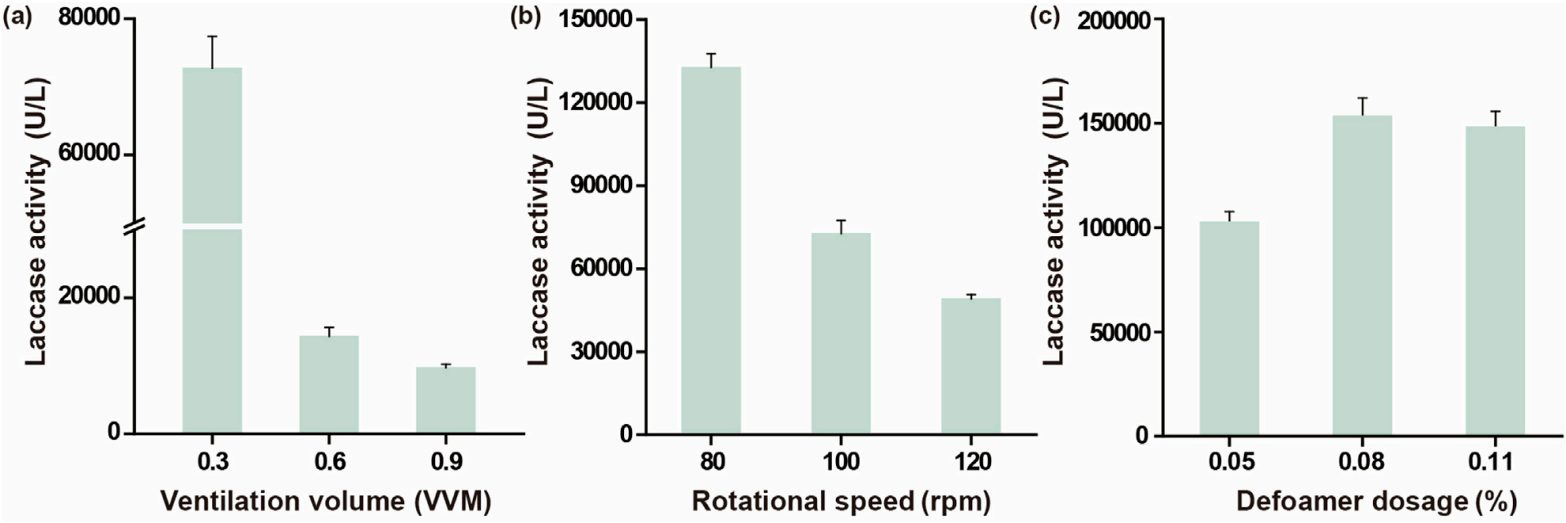
Effects of ventilation ratio, rotating speed, and defoamer dosage on laccase activity **(a)** Laccase activity with different ventilation ratios. **(b)** Laccase activity with different rotating speeds. **(c)** Laccase activity with different defoamer dosages.

During the foam-forming period of 24 h–48 h, the aeration rates were adjusted to 0.3, 0.6, and 0.9 VVM, respectively, while other fermentation parameters remained unchanged. Foam was significantly reduced at an aeration rate of 0.3 VVM, while foam remained severe at 0.6 and 0.9 VVM. The enzyme activities under these three aeration rates are shown in Figure 3. Reducing the aeration rate during this period significantly increased enzyme activity. This is because increased ventilation enhances gas-liquid contact, promoting foam formation. Reducing ventilation decreases foam formation and slightly decreases DO, but overall, reducing ventilation during this period aids microbial growth and metabolism.

During the foam-forming period of 24 h–48 h (with an aeration rate set at 0.3 VVM), the agitation speeds were varied to 80, 100, and 120 rpm. Foam decreased at 80 rpm but remained evident at 100 and 120 × *g*. The enzyme activities under these three agitation speeds are shown in Figure 3. These indicate that reducing the agitation speed significantly increases enzyme activity, with a mechanism similar to that of the aeration rate.

During the foam-forming period of 24 h–48 h, with an aeration rate of 0.3 VVM and an agitation speed of 80 rpm, silicone oil antifoam was used, and the initial antifoam dosage was adjusted to 0.05%, 0.08%, and 0.11%, respectively. No foam was observed at 0.08% and 0.11%. The enzyme activities under these three antifoam dosages are shown in Figure 3. Antifoam dosages of 0.08% and 0.11% achieved 70.5% of the enzyme activity at a liquid-loading ratio of 0.4, which is 10.5 times the enzyme activity before optimization. To conserve antifoam usage, 0.08% was selected as the optimal dosage.

### 4.5 Analysis of laccase enzymatic properties

**FIGURE 4:**
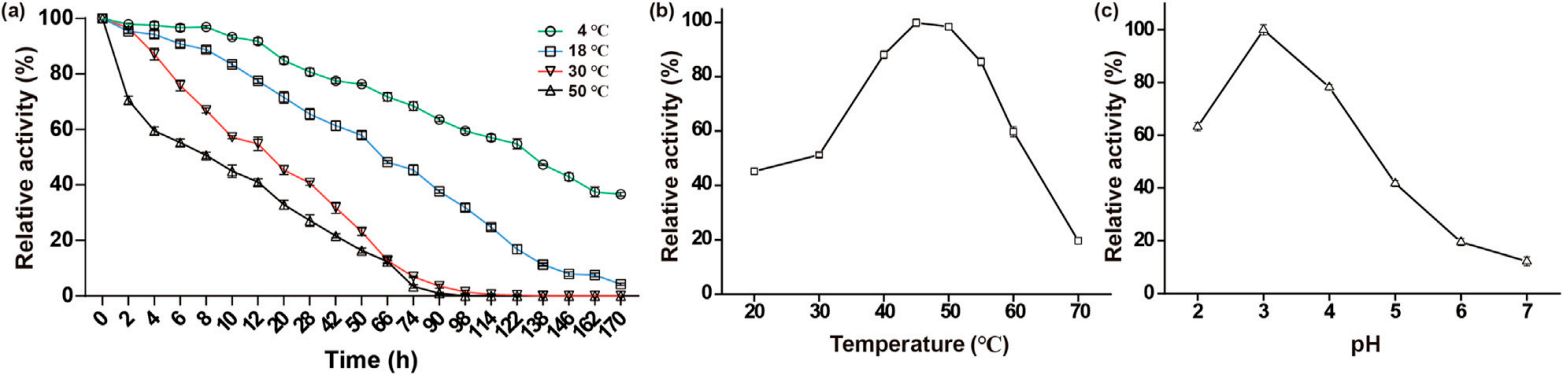
Effects of storage temperature, optimal temperature, and pH on residual activity of laccase **(a)** Effects of storage temperature on laccase activity. **(b)** Effects of optimal temperature on laccase activity. **(c)** Effects of pH on laccase activity.

The laccase enzyme activities at storage temperatures of 50, 30, 18, and 4 °C are shown in Figure 4. The residual enzyme activity decreased with prolonged storage time within the range of 4°C–50 °C, and the decrease was smaller at lower storage temperatures. Laccase activity was relatively stable under conditions of 4°C–18°C, maintaining over 70% residual activity after 20 h of storage. At 4°C, the laccase retained over 50% of relative activity even after a storage duration of 122 h. However, due to the emergence of malodorous odor at temperatures ranging from 18°C to 50 °C around the 138 h mark, the storage time experiment was only extended up to 170 h for temperature conditions. The enzyme activity changes in the fermentation broth for 0–6 months in a frozen state were determined, and laccase maintained stable activity without decrease. Although high temperatures (such as 50 °C) are more suitable for many enzymatic processes (Camassola et al., 2004), the laccase exhibited high stability, retaining over 60% of its initial activity after 3 h of storage. This characteristic is superior to that of *P. ostreatus* HP-1 basidiomycetes (Patel et al., 2014) and *Marasmiellus palmivorus* VE111.

The laccase enzyme activities at reaction temperatures of 30, 40, 50, 60, and 70 °C are shown in Figure 4. The optimal reaction temperature for laccase produced by *G. lucidum* was 45°C–50 °C. When laccase reacted at different temperatures, the enzyme activity gradually increased within the range below 40 °C and decreased above 50 °C. Similarly, Fang et al. (Fang et al., 2015) obtained maximum activity from *G. lucidum* 77,002 cultures at 45 °C.

The laccase enzyme activities at pH values of 2.0, 3.0, 4.0, 5.0, 6.0, and 7.0 are shown in Figure 4. Laccase activity was high under acidic conditions, with an optimal reaction pH of 3.0. As the pH increased, laccase activity gradually decreased, indicating that this enzyme is acidic. These data are similar to the findings of Kandasamy et al., who confirmed that the optimal reaction pH for laccase extracted from Hexagonia hirta MSF2 in the citrate-phosphate buffer is 3.4 (Kandasamy et al., 2016). In Fang et al.'s study of *G. lucidum* 77,002 (Fang et al., 2015), the optimal pH range for citrate-phosphate buffer varied from 2.5 to 5.0, depending on the substrate used.

### 4.6 TEA and LCA analysis

The cost of the fermentation medium encompasses expenses related to the medium itself, consumables, personnel, equipment depreciation, and utilities (water, electricity, gas, and steam) (Table 4). Each batch of fermentation broth can yield 1200 L × 0.6 = 720 L, herein, 1200 L represents the volume of the culture medium, and 0.6 is the liquid-filling coefficient. The total cost for each 720 L batch of fermentation broth is calculated to be 524.49 $. After filtration of the fermentation broth, the filter residue is discarded as solid waste, while the filtrate is collected for further use. Consequently, no wastewater is generated. The typical yield of the filtrate is 75%, resulting in 720 L × 75% = 540 L of filtrate per batch. When converted to a cost per gram of fermentation broth, it amounts to 0.000975 $, which is equivalent to 0.0007‰–0.0035‰ of the price of commercial laccase.

**TABLE 4:** Price analysis of batch fermentation broth.

Item	Details	Cost per 720 L batch of fermentation Broth ($)
Medium	Yeast extract 3.0 g/L, corn steep liquor 20.0 g/L, wheat bran 31.0 g/L, tobacco stem powder 10.0 g/L, potassium dihydrogen phosphate 1.1 g/L, magnesium sulfate heptahydrate 1.0 g/L, vitamin B1 0.1 g/L, antifoaming agent at a ratio of 0.08‰, glucose-potato water 2.46 g/L	241.50
Consumables	Filters, pipette tips, sealing films, kraft paperetc.	1.39
Personnel wages	2 personnel operating 5 sets of fermenters, with each fermenter undergoing a 4-day fermentation process and a 1-day cleaning process	55.72
Equipment depreciation	Shakers, clean benches, 100-L seed fermenters, 1200-L fermenters, air compressors, steam generators, with equipment depreciation calculated over a 15-year period	127.18
Water	Water for medium preparation and cleaning	0.90
Utilities (electricity, gas, and steam)	Electricity consumption by equipment and electrical control systems, electricity for compressed air production by air compressors, electricity for steam generation by steam generators, and electricity for temperature control	97.79
Total		524.49

## 5 Conclusion

Based on 200 L and 1200 L fermentation tanks, this study established a regression model for high laccase production by *G. lucidum* through Plackett–Burman, steepest ascent, and Box–Behnken response surface methodology. The optimal conditions determined were a fermentation temperature of 30°C, an aeration rate of 0.66, and an agitation speed of 100 rpm, resulting in an average laccase activity of 214,185.2 U/L in the fermentation tank. The pH of fermentation broth at different enzyme production levels showed a trend of initial stability, followed by a decrease and a subsequent increase. The higher the fermentation time corresponding to the lowest pH point, the higher the enzyme activity. Additionally, correlations between pH, DO, enzyme activity, and tank discharge signals were identified. The critical foam formation challenge was addressed by setting an aeration rate of 0.2, an agitation speed of 80 rpm, and an antifoam dosage of 0.08% during the foam-forming period of 36 h–54 h. This allowed the liquid-loading ratio to be increased to 0.6, with an enzyme activity of 153,483 U/L. Finally, the enzymatic properties of laccase were analyzed, revealing an optimal temperature range of 45°C–50 °C and an optimal pH of 3.0. Lower temperatures benefit storage.

## Data Availability

The original contributions presented in the study are included in the article/Supplementary Material, further inquiries can be directed to the corresponding authors.

## Abbreviations

SSF, Solid-state fermentation; SmF, Submerged fermentation; RSM, Response surface methodology; PDB, Potato dextrose broth; DO, Dissolved oxygen.
