# Supplementary material for: Optimization of the scale-up production process for high-yield laccase from white-rot fungi
Source: Front Bioeng Biotechnol. 2025 Aug 15;13:1631687. doi: 10.3389/fbioe.2025.1631687 (PMC12394975; doi:10.3389/fbioe.2025.1631687)
Supplement: Supplementary file 1 [file Table1.docx]

**Supplementary material**

**Optimization of the scale-up production process for high-yield laccase from white-rot fungi**

^1^Yaping Ma^1^, ^1^Minchang Liu^1^, Ruifang Gu^1^, Rongya Zhang^1^, Xiaomei Ji^2,3^, Juan Zhang^2,3^, Wu Wen^1^*, Zheng Peng ^2,3*^

^1^Technology Center, China Tobacco Sichuan Industrial Co., Ltd., Chengdu 610066, China

^2^Key Laboratory of Industrial Biotechnology, Ministry of Education, School of Biotechnology, Jiangnan University, Wuxi, China

^3^Science Center for Future Foods, Jiangnan University

^1^The authors contribute equally to the article.

*Corresponding authors: Wu Wen and Zheng Peng

E-mail addresses: wenwu@sctobacco.com, zhengpeng@jiangnan.edu.cn

**Tables**

**Table S1 Factors and levels of Plackett-Burman**

| Level | Temperature  (°C) | Ventilation volume (VVM) | Rotational speed  (rpm) | Inoculation amount  (%) | Medium volume |
| --- | --- | --- | --- | --- | --- |
| -1 | 26 | 0.5 | 50 | 7 | 0.4 |
| 1 | 34 | 0.8 | 100 | 13 | 0.6 |

**Table S2 Plackett-Burman test**

| No. | Temperature  (°C) | Ventilation volume (VVM) | Rotational speed  (rpm) | Inoculation amount  (%) | Medium volume | Laccase activity (U/L) |
| --- | --- | --- | --- | --- | --- | --- |
| 1 | 34 | 0.8 | 100 | 7 | 0.4 | 127542 |
| 2 | 26 | 0.5 | 100 | 7 | 0.6 | 16691 |
| 3 | 34 | 0.8 | 50 | 7 | 0.4 | 84351 |
| 4 | 26 | 0.5 | 50 | 13 | 0.4 | 144397 |
| 5 | 26 | 0.5 | 50 | 7 | 0.4 | 146319 |
| 6 | 26 | 0.8 | 100 | 7 | 0.6 | 10576 |
| 7 | 26 | 0.8 | 100 | 13 | 0.4 | 169415 |
| 8 | 34 | 0.8 | 50 | 13 | 0.6 | 10684 |
| 9 | 34 | 0.5 | 50 | 7 | 0.6 | 13658 |
| 10 | 34 | 0.5 | 100 | 13 | 0.6 | 7703 |
| 11 | 34 | 0.5 | 100 | 13 | 0.4 | 122481 |
| 12 | 26 | 0.8 | 50 | 13 | 0.6 | 13590 |

**Table S3 Factors and levels of Box-Behnken**

| **Level** | **Factors** | | |
| --- | --- | --- | --- |
|  | **A: Temperature (°C)** | **B: Ventilation volume (VVM)** | **C: Rotational speed (rpm)** |
| -1 | 32 | 0.8 | 100 |
| 0 | 30 | 0.6 | 80 |
| 1 | 28 | 0.4 | 60 |

**Table S4 Box-Behnken in terms of experimental results**

| Test | Temperature (°C) | Ventilation volume (VVM) | Rotational speed (rpm) | Laccase activity (U/L) |  |
| --- | --- | --- | --- | --- | --- |
| 1 | 32 | 0.6 | 100 | 168978 | |
| 2 | 30 | 0.4 | 100 | 170197 | |
| 3 | 30 | 0.8 | 100 | 217724 | |
| 4 | 28 | 0.4 | 80 | 76315 | |
| 5 | 28 | 0.6 | 60 | 80356 | |
| 6 | 32 | 0.4 | 80 | 93568 | |
| 7 | 32 | 0.6 | 60 | 83716 | |
| 8 | 28 | 0.6 | 100 | 153581 | |
| 9 | 28 | 0.8 | 80 | 132568 | |
| 10 | 30 | 0.8 | 60 | 123463 | |
| 11 | 30 | 0.4 | 60 | 108550 | |
| 12 | 32 | 0.8 | 80 | 92376 |  |
